# Polyarteritis nodosa in a patient with chronic hepatitis B following COVID-19 vaccination: a case report

**DOI:** 10.1093/omcr/omad092

**Published:** 2023-09-25

**Authors:** Aseel Abuhammad, Osama N Dukmak, Diya Asad, Izzeddin A Bakri, Saed I Y Attawna

**Affiliations:** Al-Quds University Faculty of Medicine, Jerusalem, State of Palestine; Al-Quds University Faculty of Medicine, Jerusalem, State of Palestine; Al-Quds University Faculty of Medicine, Jerusalem, State of Palestine; Al-Ahli Hospital, Hebron, State of Palestine; Al-Ahli Hospital, Hebron, State of Palestine

## Abstract

Different types of vasculitis have been reported after various vaccine administrations. Recently, the coronavirus disease 2019 (COVID-19) vaccine was one of the most common vaccine-induced vasculitis. Herein, we describe a 56-year-old male patient with chronic hepatitis B who presented with abdominal pain for 2 days, which was associated with vomiting and bloody diarrhea. He had a history of petechial rash for 25 days, multiple joint pain and lower limb weakness after the second dose of the COVID-19 vaccine. A skin biopsy showed medium-sized vessel vasculitis. Polyarteritis nodosa (PAN) was diagnosed depending on the American College of Rheumatology criteria. He was treated with steroids, plasmapheresis and antiviral medication with a good prognosis. In patients with a past medical history of chronic hepatitis B, the covid vaccine may be associated with an increased risk of developing a PAN, so clinicians should suspect the occurrence of this disease after COVID-19 vaccination.

## INTRODUCTION

Polyarteritis nodosa (PAN) is a systemic necrotizing vasculitis affecting medium-sized vessels, which include the main visceral arteries and their branches [1, 2]. Multiple case reports have suggested a link between PAN and vaccination, particularly hepatitis B vaccination [3]. However, PAN, in association with the coronavirus disease 2019 (COVID-19) vaccine, was relay reported.

COVID-19 vaccines were given to most people (all over the world) to reduce and prevent the rapid spread of the Coronavirus infection. Multiple vaccines were approved, which include the mRNA vaccines (Pfizer-BioNTech or mRNA-Moderna), viral vector vaccines (Johnson & Johnson or Oxford) and inactivated viral vaccines. Although these vaccines have been effective and safe in most conditions, different drug reactions were reported, such as vasculitis [4, 5].

We present a case of PAN following COVID-19 vaccination in a male patient with a medical history of chronic hepatitis B. This case has been reported in line with Care criteria (see Methods section).

## CASE REPORT

A 56-year-old male patient with a known history of hypertension presented with diffuse colicky abdominal pain for 2 days, which was associated with vomiting and bloody diarrhea. He had a history of petechial rash for 25 days, which started after the second dose of the COVID-19 vaccine in both lower limbs and then spread rapidly to cover all over his body over a few days. The rash was tender to the touch, not blanching and was associated with multiple joint pain.

The patient had a history of hepatitis B for >24 years and was on lamivudine at the first 5 years of diagnosis. Eight days after receiving the second dose of covid vaccine (Pfizer), he developed his symptoms.

On examination, the patient appeared to be oriented, alert, afebrile and in no respiratory distress. His blood pressure was high (180/87 mm Hg), and his heart rate (87), oxygen saturation (95%) and temperature (36.5) were within normal limits. There were purpuric non-blanching 1–2-cm multiple lesions covering the trunk, upper and lower limbs (Fig. 3).

Laboratory data were notable for a positive fecal occult blood test and (2+) hematuria on urine dip, with 35–40 red blood cells/high-power field, 3 white blood cells/high-power field, moderate bacteria in the urine sediment and no proteinuria. Complete blood count showed normocytic anemia with Hb of 8.4 and mean corpuscular volume of 89, normal WBC at 8 × 10^3/ml and thrombocytopenia at 90 × 103/ml. Blood urea nitrogen of 21 mg/dl, a creatinine of 1.35 mg/dl, AST of 40 U/l, ALT of 42 U/l, INR of 1.13, amylase of 59 U/l, C3 of 54 mg/dl and CPR of 50 mg/l. Antineutrophil cytoplasmic antibodies test and ANA were negative. HBeAg and hepatitis B virus (HBV) deoxyribonucleic acid polymerase chain reaction tests were positive with negative HBeAb.

**Figure 1:**
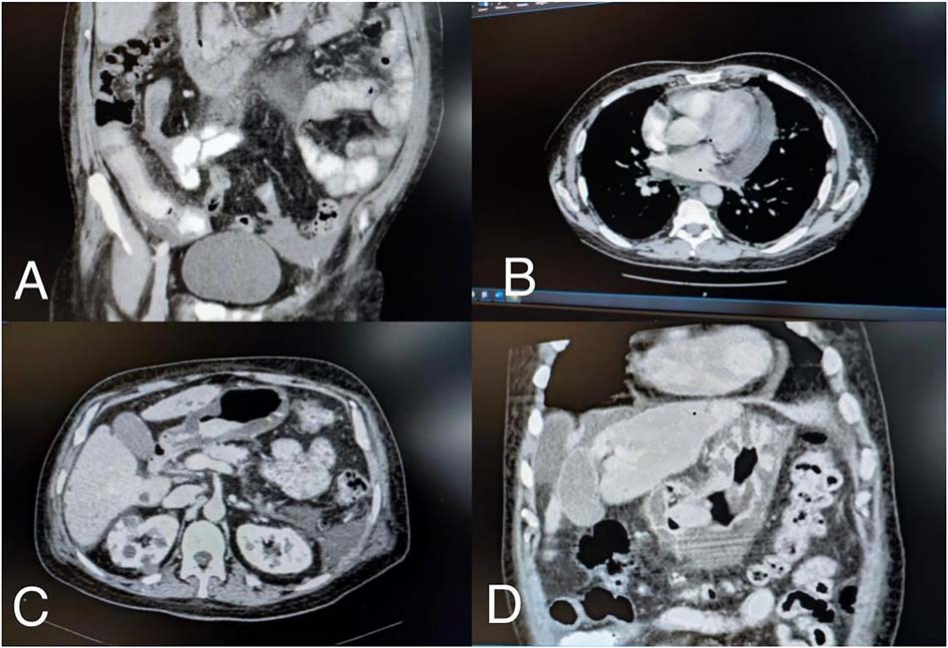
(**A**) Small bowel loops with submucosal edema, (**B**) pericardial effusion, (**C**) and (**D**) mild wall thickening with submucosal edema at the pylorus.

Abdomen computed tomography angiogram showed thickened enhancing wall of the small bowel loops, with submucosal edema associated with stranding of the surrounding fatty planes, mild-to-moderate free fluid and mild wall thickening with submucosal edema of the stomach mainly at the pylorus (Fig. 1).

**Figure 2:**
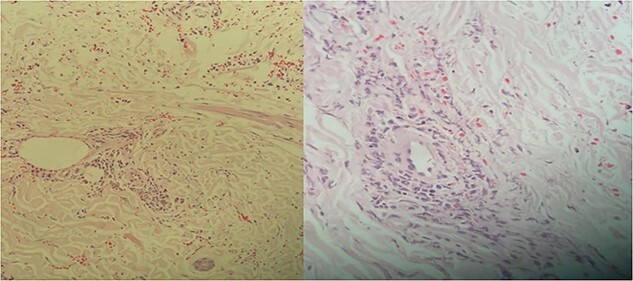
Mild-to-moderate lymphocytic infiltrate around the medium-sized arteries in the junction of dermis and subcutis; the appearance is most suggestive of medium-sized vessel vasculitis.

**Figure 3:**
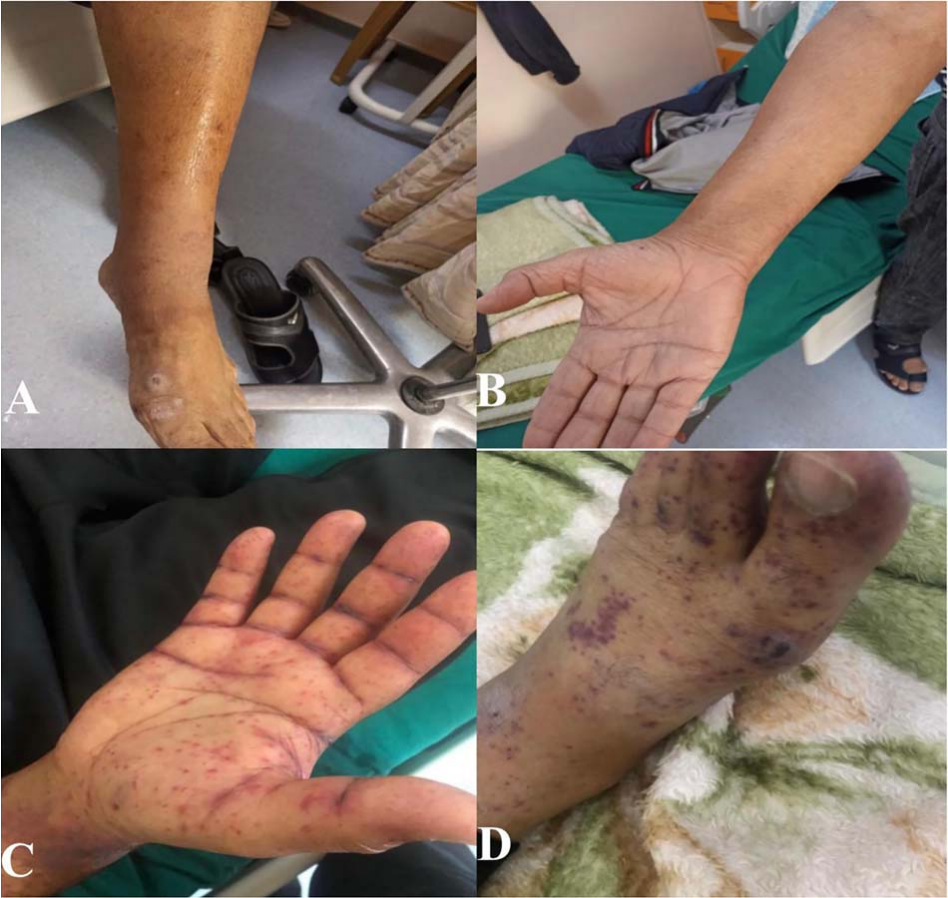
(**A**, **B**) Complete resolution of the rash after treatment with plasmapheresis; (**C**, **D**) purpuric rash in the lower and upper limbs.

Due to lower limb weakness, numbness and limitation of daily activity, a nerve conduction study (NCS) was requested to look for any evidence of neuropathic changes. NCS showed evidence of demyelinating process (with temporal dispersion sign), affecting lower limb nerves more than upper limb tested nerves. In light of the clinical picture, this study suggested acquired sensory motor neuropathy with secondary axonal injury. The patient’s clinical presentation was suggestive of PAN, and this diagnosis was confirmed on skin biopsy, which showed a medium-sized vessel vasculitis (Fig. 2). He was treated with prednisone 60 mg tapered over 2 weeks. After six sessions of plasmapheresis, his skin rash disappeared completely, but he continued to have positive HBeAg positive and negative HBeAb. For that, he continued the plasmapheresis for a total of 17 sessions of plasmapheresis until seroconversion occurred (negative HBeAg and positive HBeAb). In addition, he was also treated with tenofovir 300 mg daily (Fig. 3).

## DISCUSSION

PAN is a rare type of vasculitis that affects men more than women by around (1.5:1) and is associated with HBV infection, which typically occurs before PAN disease in a short period. Moreover, the late onset of PAN was rare [2, 6]. For that reason, another causative agent could be present, for example, the new viral infection. Guillevin L. *et al*. [6] In our patient, who had a chronic hepatitis B, PAN developed soon after vaccination. This finding suggests a possible pathogenetic link between these co-factors.

Our patient presented with abdominal pain, a non-blanching petechial rash, joint pain, neurological symptoms, a history of hepatitis B and elevated creatinine. In light of the resulting biopsy, we suspected PAN depending on American College of Rheumatology criteria (ACR criteria) for PAN. Yamamoto S. *et al*. [7] (Table 1).

**Table 1:** ACR criteria 1990 for PAN

Classification criteria	Case fulfillment
Weight loss > 4 KG	−
Livedo reticularis	−
Testicular pain	−
Myalgia, weakness, leg tenderness	+
Mono/polyneuropathy	+
DBP > 90 mmHg	−
Elevated blood urea, creatinine	+
Hepatitis B	+
Arteriographic abnormality	−
Biopsy showing polymorphonuclear cells	+

To the best of our knowledge, only three cases were reported with medium-vessel vasculitis related to COVID-19 vaccination. The first one was for a 46-year- an old male patient, who presented with vasculitis that affected the celiac trunk and its branches after 1 week following the second dose of the Pfizer COVID-19 vaccine, which was diagnosed by clinical and radiographic manifestation with medium-vessel vasculitis. Al-Allaf A. W. *et al*. [5] The second patient was a 41-year-old female who presented with fever and myalgia after 35 days following the second dose of the Moderna vaccine. Ohmura S. I. *et al*. [4]

The last patient was a 73-year-old male known history of chronic hepatitis B, who presented with fever, arthralgia, purpura, renal failure and orchitis after the first dose of the COVID-19 vaccine. A kidney biopsy showed medium-vessel vasculitis. Fillon A. *et al*. [8]

In conclusion, any patients with a history of hepatitis B infection presented with rash, acute renal failure and other symptoms related to COVID-19 immunization should be evaluated to rule out PAN vasculitis. When that occurs, treatment with an antiviral strategy should be combined with treatment of other etiologies.

## REGISTRATION OF RESEARCH STUDIES

N/A.

## METHODS

This case has been reported in line with Care criteria [9].

## References

[1] Hernández-Rodríguez J, Alba MA, Prieto-González S, Cid MC. Diagnosis and classification of polyarteritis nodosa. J Autoimmun 2014;48-49:84–9. 10.1016/j.jaut.2014.01.029. 24485157

[2] Hočevar A, Tomšič M, Perdan Pirkmajer K. Clinical approach to diagnosis and therapy of polyarteritis nodosa. Curr Rheumatol Rep 2021;23:14. Published 2021 Feb 10. 10.1007/s11926-021-00983-2. 33569653

[3] Begier EM, Langford CA, Sneller MC, Wise RP, Ball R, VAERS Working Group. Polyarteritis nodosa reports to the vaccine adverse event reporting system (VAERS): implications for assessment of suspected vaccine-provoked vasculitis. J Rheumatol 2004;31:2181–8. 15517631

[4] Ohmura SI, Ohkubo Y, Ishihara R, Otsuki Y, Miyamoto T. Medium-vessel vasculitis presenting with myalgia following COVID-19 Moderna vaccination. Intern Med 2022;61:3453–7. 10.2169/internalmedicine.0293-22. 36070946 PMC9751726

[5] Al-Allaf AW, Razok A, Al-Allaf Y, Aker L. Post-COVID-19 vaccine medium-vessel vasculitis and acute anterior uveitis, causation vs temporal relation; case report and literature review. Ann Med Surg (Lond) 2022;75:103407. 10.1016/j.amsu.2022.103407. 35228869 PMC8867999

[6] Guillevin L, Mahr A, Callard P, Godmer P, Pagnoux C, Leray E et al. Hepatitis B virus-associated polyarteritis nodosa: clinical characteristics, outcome, and impact of treatment in 115 patients. Medicine (Baltimore) 2005;84:313–22. 10.1097/01.md.0000180792.80212.5e. 16148731

[7] Yamamoto S, Oiwa H. Provisional seven-item criteria for the diagnosis of polyarteritis nodosa. Rheumatol Int 2020;40:1223–7. 10.1007/s00296-020-04535-2. 32107599

[8] Fillon A, Sautenet B, Barbet C, Moret L, Thillard EM, Jonville-Béra AP et al. *De novo* and relapsing necrotizing vasculitis after COVID-19 vaccination. Clin Kidney J 2022;15:560–3 Published 2021 Dec 20. 10.1093/ckj/sfab285. 35211310 PMC8862065

[9] Gagnier JJ, Kienle G, Altman DG, Moher D, Sox H, Riley D et al. The CARE guidelines: consensus-based clinical case reporting guideline development. BMJ Case Rep 2013;2013:bcr2013201554. 10.1136/bcr-2013-201554. PMC3844611 24228906

